# Wild bird‐associated *C* *ampylobacter jejuni* isolates are a consistent source of human disease, in Oxfordshire, United Kingdom

**DOI:** 10.1111/1758-2229.12314

**Published:** 2015-09-08

**Authors:** Alison J. Cody, Noel D. McCarthy, James E. Bray, Helen M. L. Wimalarathna, Frances M. Colles, Melissa J. Jansen van Rensburg, Kate E. Dingle, Jonas Waldenström, Martin C. J. Maiden

**Affiliations:** ^1^ Department of ZoologyUniversity of OxfordOxfordUK; ^2^ Health Protection AgencyLondonUK; ^3^ Warwick Medical SchoolUniversity of WarwickCoventryUK; ^4^ NIHR Health Protections Research Unit in Gastrointestinal InfectionsUniversity of OxfordOxfordUK; ^5^ Nuffield Department of Clinical MedicineOxford UniversityJohn Radcliffe HospitalOxfordUK; ^6^ Centre for Ecology and Evolution in Microbial Model SystemsLinnaeus UniversityKalmarSweden

## Abstract

The contribution of wild birds as a source of human campylobacteriosis was investigated in Oxfordshire, United Kingdom (UK) over a 10 year period. The probable origin of human *C* *ampylobacter jejuni* genotypes, as described by multilocus sequence typing, was estimated by comparison with reference populations of isolates from farm animals and five wild bird families, using the STRUCTURE algorithm. Wild bird‐attributed isolates accounted for between 476 (2.1%) and 543 (3.5%) cases annually. This proportion did not vary significantly by study year (*P* = 0.934) but varied seasonally, with wild bird‐attributed genotypes comprising a greater proportion of isolates during warmer compared with cooler months (*P* = 0.003). The highest proportion of wild bird‐attributed illness occurred in August (*P* < 0.001), with a significantly lower proportion in November (*P* = 0.018). Among genotypes attributed to specific groups of wild birds, seasonality was most apparent for *T* *urdidae*‐attributed isolates, which were absent during cooler, winter months. This study is consistent with some wild bird species representing a persistent source of campylobacteriosis, and contributing a distinctive seasonal pattern to disease burden. If Oxfordshire is representative of the UK as a whole in this respect, these data suggest that the national burden of wild bird‐attributed isolates could be in the order of 10 000 annually.

## Introduction

*Campylobacter jejuni* and *Campylobacter coli* are widespread intestinal commensals that rarely appear to cause disease in their various wild and domesticated bird and animal hosts (Humphrey *et al*., 2014); however, they are a frequent zoonotic cause of bacterial gastroenteritis in humans. During 2012, 71 365 cases of human campylobacteriosis were reported in the United Kingdom (UK) (HPS, 2013; PHE, 2013b), of which approximately 90% of cases were caused by *C. jejuni* (Gillespie *et al*., 2006; Cody *et al*., 2012). Estimates of seven additional affected individuals for each diagnosed and reported case means that the actual burden of this disease is much higher (Tam *et al*., 2012), so that even infection sources causing a minor percentage of total infections are of public health importance.

Multilocus sequence typing (MLST) studies of *C. jejuni* have described a genetically diverse, essentially non‐clonal population, which contains a large number of genotypically related groups of isolates, known as clonal complexes (ccs) (Dingle *et al*., 2002). Some genotypes are characteristically associated with particular isolation sources (Dingle *et al*., 2002; McCarthy *et al*., 2007). Exploitation of these insights in host association studies has identified both multihost and host‐associated ccs, with some genotypes common to farm animals and poultry. Further, phylogenetically distinct *C. jejuni* have been identified in different species of wild animals and birds, with host species association particularly strong among wild birds (Dingle *et al*., 2002; Sheppard *et al*., 2009a; Strachan *et al*., 2009; Williams *et al*., 2010; Griekspoor *et al*., 2013). The association between *Campylobacter* genotype and host species exceeds that between genotype and geographic origin (Sheppard *et al*., 2010; Griekspoor *et al*., 2013).

Source attribution analyses have implicated contaminated poultry meat as the major cause of clinical infection in the UK and elsewhere, by either consumption of undercooked meat or by cross‐contamination of other foodstuffs (Mullner *et al*., 2009a; 2010; Sheppard *et al*., 2009b; Strachan *et al*., 2009; Domingues *et al*., 2012; Mughini Gras *et al*., 2012; Kittl *et al*., 2013; Levesque *et al*., 2013). These studies do not, however, exclude a role for bovine, ovine and other sources in human disease (Mullner *et al*., 2009b; Sheppard *et al*., 2009b; Strachan *et al*., 2009). These alternative sources include wild birds via faecal contamination of equipment and surfaces in children's playgrounds (French *et al*., 2009), consumption of milk from bottles with bird‐pecked tops (Southern and Kutscher, 1987; Riordan *et al*., 1993) or by environmental exposure during leisure or employment activities (Strachan *et al*., 2009).

Human campylobacteriosis rates show distinct seasonality in temperate climates demonstrating a summer peak in disease incidence (Nylen *et al*., 2002; Kovats *et al*., 2005). In countries with less severe winters, this occurs 3 months prior to the peak in environmental temperature (Kovats *et al*., 2005) and is evident earlier in the year in children under 5 years of age living in rural areas than in adults (Strachan *et al*., 2013). The peak observed among young children has different predominant attribution sources in rural and urban areas, with the majority of urban cases having chicken‐associated genotypes whereas those living in rural locations are apparently infected more by ruminant and wild‐bird attributed genotypes (Strachan *et al*., 2009). Here we investigate the contribution of wild bird isolates to the incidence of human campylobacteriosis among patients of all age groups using 10 years of continuous *Campylobacter* genotype surveillance in Oxfordshire, United Kingdom, and a replicate analysis using 3 years of equivalent data from patients in Nottinghamshire and Hampshire. Such large datasets, combined with the strong association of *Campylobacter* genotype with wild bird host (Griekspoor *et al*., 2013), enable a robust estimation of the contribution made by this infection source to the burden of human disease.

## Results and discussion

### MLST of Oxfordshire human isolates

**Figure 1:**
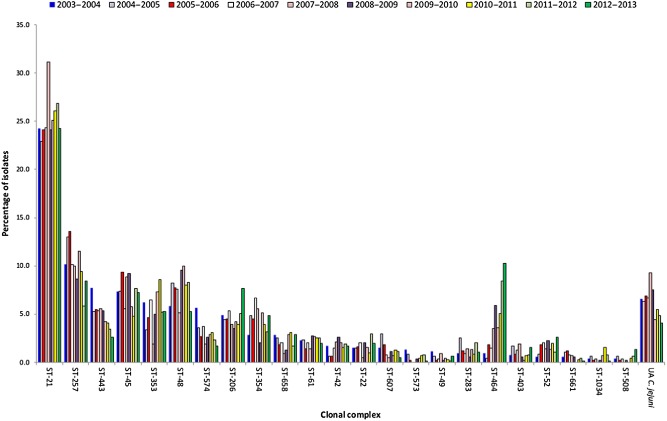
Comparison of the proportion of clonal complexes identified from human campylobacteriosis isolates (*n* = 5628) from Oxfordshire, UK, between September 2003 and September 2013. A further 13 clonal complexes were represented by less than 1% of isolates in any year. Multilocus sequence typing (MLST) of single colonies isolated prior to June 2011 was performed as previously described (Cody *et al*., 2012), whereas MLST data from subsequent isolates were extracted from whole genome sequence (Cody *et al*., 2013).

Between September 2003 and September 2013, complete MLST profiles were obtained for 5628 human campylobacteriosis isolates from Oxfordshire, UK. The seven most predominant ccs across all study years were; ST‐21 cc; ST‐257 cc; ST‐443 cc; ST‐45 cc; ST‐353 cc, ST‐48 cc and ST‐206 cc; together with sequence types (STs) unassigned to any cc (Fig. 1). This distribution of complexes reflects that seen elsewhere in the UK and confirms the findings of previous studies that demonstrate the capacity of a single sentinel site to accurately reflect national epidemiology (Dingle *et al*., 2008; McCarthy *et al*., 2012). The overall changes in prevalence observed in this study for four chicken‐associated ccs (ST‐257, ST‐443, ST‐464 and ST‐574) possibly reflect the dynamics of these genotypes in poultry flocks.

### Attribution analysis of human isolates

**Figure 2:**
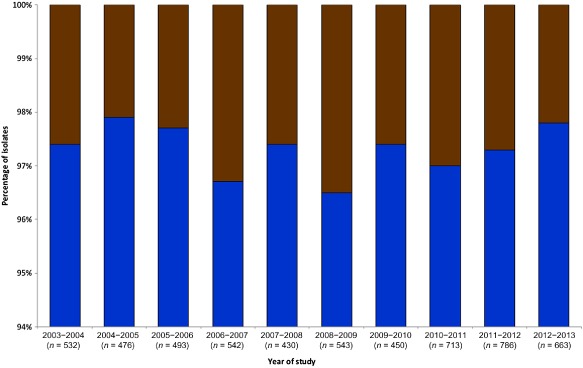
Proportion of clinical isolates collected in Oxfordshire, UK, between September 2003 and September 2013 (*n* = 5618), attributed to farm animals (*blue)* or wild bird sources (*brown*). Farm reference data sets consisted of 1541 UK isolates from 742 retail poultry (2001–2005), 582 cattle and 217 sheep (2003–2006) which included isolates from Sheppard and colleagues (2009b; 2010). Wild bird reference datasets totalled 921 isolates from the UK (2002–2005) (Colles *et al*., 2008b; 2009), Australia (2004–2006) (Hansbro *et al*., 2010) and Sweden (1999–2002) (Broman *et al*., 2002; 2004; Waldenstrom *et al*., 2002). All referenced and additional data were accessed via the isolate database at http://pubmlst.org/campylobacter (Table S1). The frequency of MLST genotypes from different host species was calculated using the STRUCTURE algorithm (Pritchard *et al*., 2000), to which isolates of unknown source were assigned. All genotypes were identified by the USEPOPINFO flag, frequencies between populations regarded as independent and a no‐admixture model assumed. Assignments were performed using 1000 burn‐in cycles and sampling 10 000 iteration cycles. Variation between years was assessed using Pearson's chi‐square statistic, or, where sample sizes were small, Fisher's exact test using Stata IC 10 (StataCorp LP, Texas).

**Figure 3:**
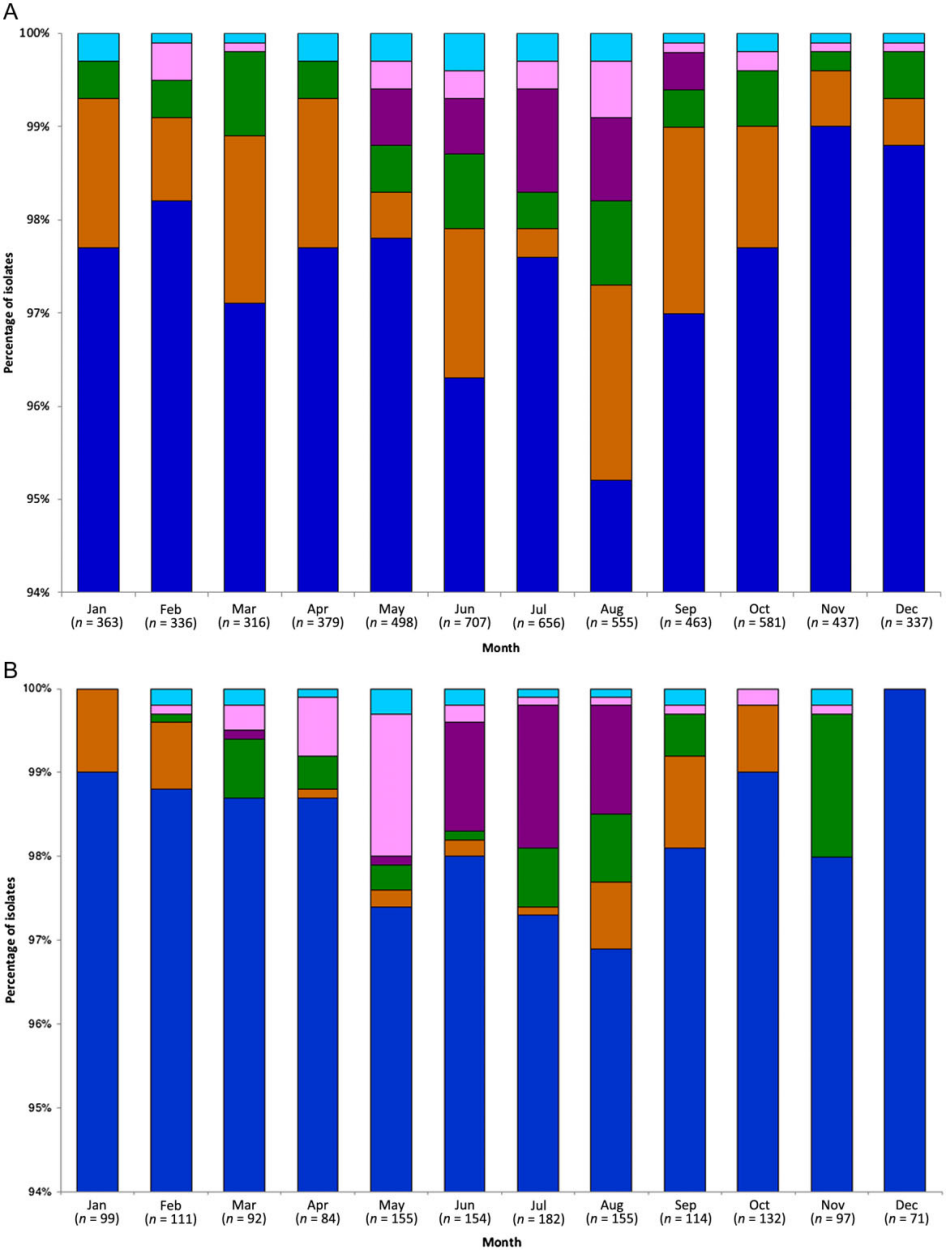
Proportion of clinical isolates attributed using STRUCTURE to farm animals (*blue*) or wild bird sources: *Anatidae* (mallards, geese) brown; *Laridae* (gulls) green; *Turdidae* (blackbirds, song thrush) purple; *Sturnidae* (starlings) pink; *Scolopacidae* (dunlin, sharp‐tailed sandpipers) turquoise, was performed as described above, by month. A. Oxfordshire, UK, between September 2003 and September 2013 (*n* = 5618). B. Hampshire and Nottinghamshire, UK, between May 2000 and April 2003 (*n* = 1446).

The proportion of clinical isolates from Oxfordshire attributed to wild birds, 2.1%–3.5% annually was similar to values for other human populations reported by two smaller studies (Sheppard *et al*., 2009b; Levesque *et al*., 2013), and showed no consistent secular trend and no statistically significant variation between years (*P* = 0.934) (Fig. 2). However, there was a significant variation in the percentage of these human cases attributable to wild birds during cooler (October to April) relative to the warmer (May to September) months (*P* = 0.003) (Fig. 3), with individuals more likely to acquire a wild bird *C. jejuni* genotype during August (*P* < 0.001) and less likely to do so during November (*P* = 0.018) than during other months of the year. Among the five groups of wild birds a seasonal pattern was observed for Oxfordshire disease isolates attributable to members of the *Turdidae* (blackbirds and song thrushes), which accounted for between 0.4% and 1.1% of cases in the warmer months (May to September), but no disease during cooler periods (October to April) (*P* = 0.011). In contrast, cases attributed to members of the *Anatidae* (mallards and geese) and *Laridae* (gulls) occurred across all months, without evidence for seasonal variation (Fig. 3). Cases attributable to birds of the *Scolopacidae* (dunlin and sandpipers) and *Sturnidae* (starlings) were fewer in number not allowing inference for or against seasonal patterns.

Human disease isolate data from Hampshire and Nottinghamshire between May 2000 and April 2003 (McCarthy *et al*., 2012) showed a similar pattern of attribution with respect to wild bird genotypes, which included a statistically significant difference in wild bird‐associated seasonal signal (*P* = 0.049) between warmer and cooler months (Fig. 3). Clinical campylobacteriosis cases attributable to members of the *Turdidae* again showed a seasonal pattern with the highest number of cases occurring in July (1.7%), June (1.3%), August (1.3%), May and March (0.1%); with no cases detected during the rest of the year. Lower numbers of isolates contained in the smaller replicate dataset from Hampshire and Nottinghamshire limited formal inference on potential differences between different groups of birds.

The seasonality of human infection observed for wild bird attributed cases peaked in August, which is later than the seasonal peak from all sources (Nylen *et al*., 2002; Kovats *et al*., 2005; Cody *et al*., 2012). However, the observed seasonal pattern in *Turdidae* attributed clinical disease correlated inversely with the population size of this bird family, which for both species increase in late autumn and winter months by the influx of migratory birds from other populations in Northern Europe. Habitats favoured by members of the *Turdidae* include parks and gardens, which could explain direct human transmission via faecal contamination during the seasons when people are more likely to frequent these settings. Investigations specific to the acquisition of disease in young children have concluded that indirect exposure to surfaces contaminated with starling faeces (French *et al*., 2009), combined with frequent hand‐to‐mouth behaviour (Tulve *et al*., 2002), are key risk factors. In contrast, the predominant acquisition pathway of chicken‐associated genotypes appears to be via consumption of contaminated foodstuffs; risk factors for ruminant‐associated STs have included occupational and environmental exposures (Strachan *et al*., 2009; Mughini Gras *et al*., 2012).

As 71 365 human campylobacteriosis cases were reported in the UK in 2012 (HPS, 2013; PHE, 2013b), and estimates suggest that only one in seven individuals with campylobacteriosis are diagnosed and reported (Tam *et al*., 2012), even the lower estimate of 2.1% would indicate that 10 000 human campylobacteriosis cases annually in the UK are wild bird associated. These findings indicate that although wild birds are the source of a relatively small proportion of human infection, the overall disease burden from this source is substantial in absolute terms.

The presence of genotypes common to both retail poultry and wild birds was assessed; a total of 442 STs were identified in the two datasets (192 retail poultry, 250 wild bird) of which 12 (2.8%) were shared (Table S2); eight were principally identified in the poultry dataset and four predominated in wild birds. Identification of these shared STs reflects published data that show 3% and 5.9% of wild bird STs have common genotypes with those from farm animal and chicken datasets (Sheppard *et al*., 2011; Levesque *et al*., 2013). In total, the shared genotypes were represented by 48 (5.2%) of the wild bird isolates analysed in this study, of which 13 (1.4%) were from *Turdidae* species. The observed seasonality of *Turdidae*‐associated genotypes in human disease is therefore most likely due to environmental acquisition routes.

The contribution made by farm animal‐associated *Campylobacter* genotypes, particularly chicken, to human infection has been well documented (Wilson *et al*., 2008; Mullner *et al*., 2009b; Sheppard *et al*., 2009b), but there have been fewer studies which have assessed that made by wild bird genotypes (Colles *et al*., 2008a; 2009; 2011; French *et al*., 2009; Strachan *et al*., 2009; Griekspoor *et al*., 2013). The combination of information on wild bird *Campylobacter* genotypes, human campylobacteriosis genotype data from a large, longstanding, longitudinal population based surveillance programme, and the large and growing global resource of host animal reference datasets, enabled an estimate of the burden of human *Campylobacter* infection associated with wild birds. In addition, these data facilitated the investigation of temporal and wild bird species‐associated human disease patterns. These estimates were validated by repeating the analysis using a second human *Campylobacter* dataset (McCarthy *et al*., 2012).

Whilst many studies are currently focussed on the control of chicken‐acquired human disease, the number of clinical cases could be reduced by addressing transmission routes from these proportionally smaller sources of infection, which cause higher illness levels in the UK than *Salmonella* species (PHE, 2013a). Assuming the data reported here to be representative of the UK as a whole, as many as 100 000 human *Campylobacter* infections may be attributable to wild bird sources over the 10 years of this study.

## Supporting information


**Table S1.** Oxfordshire clinical, farm and wild bird *Campylobacter jejuni* isolates.
**Table S2.** Sequence types (ST) shared between retail poultry (*n* = 192) and wild bird datasets (*n* = 250).
